# Evaluation of zinc effect on cadmium action in lipid peroxidation and metallothionein levels in the brain

**DOI:** 10.1016/j.toxrep.2015.05.014

**Published:** 2015-06-05

**Authors:** Marcos M. Braga, Tuiskon Dick, Diogo L. de Oliveira, Adriele Scopel-Guerra, Ben Hur M. Mussulini, Diogo O. Souza, João Batista T. da Rocha

**Affiliations:** a Departamento de Bioquímica, Instituto de Ciências Básicas da Saúde, Universidade Federal do Rio Grande do Sul, CEP 90035-003 Porto Alegre, RS, Brazil; b Departamento de Bioquímica e Biologia Molecular, Centro de Ciências Naturais e Exatas, Universidade Federal de Santa Maria, Santa Maria, RS 97105-900, Brazil

**Keywords:** Cadmium, Zinc, Metallothionein, δ-Aminolevulinate dehydratase, Food intake

## Abstract

Cadmium (Cd) is a known hepato- and nephrotoxic pollutant and zinc (Zn) metalloproteins are important targets of Cd. Hence, the administration of Zn may mitigate Cd toxic effects. However, the interaction of Cd and Zn has been little investigated in the brain. Previously, we reported a protective effect of Zn on mortality caused by Cd in rats. Here, we tested whether the protective effect of Zn could be related to changes in brain Zn-proteins, metallothionein (MT) and δ-aminolevulinate dehydratse (δ-ALA-D). Male adult rats were daily administered for 10 days with Zn (2 mg kg^−1^), Cd (0.25 and 1 mg kg^−1^) and 0.25 mg kg^−1^ of Cd plus Zn and 1 mg kg^−1^ of Cd plus Zn. The body weight loss, food intake deprivation, and mortality occurred in 1 mg kg^−1^ of Cd, but Zn co-administration did mitigate these effects. The brain Zn content was not modified by treatment with Cd, whereas cerebral Cd levels increased in animals exposed to Cd. The administration of 0.25 mg kg^−1^ of Cd (with or without Zn) induced lipid peroxidation and decreased MT concentration, but 2 mg kg^−1^ of Zn and 1 mg kg^−1^ of Cd did not change these parameters. Brain δ-ALA-D was not modified by Cd and/or Zn treatments. Since the co-administration of Zn did not attenuate the changes induced by Cd in the brain, our results suggest that the protective effect of Zn on impairments caused by Cd in animal status is weakly related to a cerebral interaction of these metals.

## 1 Introduction

The metal cadmium (Cd) is a widespread environmental contaminant [1] and a potential toxin that may harmfully affect human health. Cadmium has a long biological half-life [2] and testicular, hepatic and renal cells are particularly sensitive to exposure to Cd [3], [4], [5], [6]. Accordingly, Cd intoxication has been associated with reactive species over production, mitochondrial injury, lipid peroxidation and cellular death in liver [7], [8] and kidney [9].

Cadmium can have also neurotoxic effects. In several experimental models, it has been reported that Cd induces oxidative damage in brain mitochondria [10], [11], impairs synaptic transmission [12], [13], and reduces neuronal differentiation and axonogenesis [14]. Thus, occupational exposure to Cd have been linked to motor and memory deficiencies [15].

The molecular toxicity of Cd is mainly related to direct action in zinc (Zn)-dependent biological pathways [16]. Cadmium exposure is able to alter Zn transporters expression in zebrafish [17] and cause Zn accumulation in different tissues of rodents [16], [18]. In addition, the Zn-dependent activity of the δ-aminolevulinate dehydratase (δ-ALA-D) is a potential target of Cd, which can replace Zn of the active site of the mammalian enzyme [19], [20], [21]. The expression of metallothionein (MT), an important protein in Zn homeostasis, has been also induced after Cd administration [22]. Overall, Cd effects may be mitigated by Zn, including Cd-induced oxidative stress [23]. The antagonist effect of Zn on Cd toxicity has led to proposition that Cd is an antimetabolite of Zn [16]. However, the interaction between Cd and Zn after systemic exposure has been little explored in the brain.

Zinc has important role in the brain and could be a highly sensitive target to action of Cd. There are a considerable number of proteins that bind to Zn in the central nervous system (CNS), for instance, δ-ALA-D [24] and MTs [25]. Moreover, a brain fraction of Zn, named chelatable Zn pool, is found free or loosely bound to biomolecules, and it is localized abundantly in synaptic vesicles [26]. This indicates that Zn participates in the regulation of synaptic physiology. As corollary, we can suppose that the interaction between Cd and Zn could be more critical in the brain.

The aim of this work was to investigate whether the protective effect of Zn on systemic effects of Cd could be related to changes in brain Zn-proteins, similar with those observed in peripheral tissues of adult rats [27], [28], [29]. Thus, the animal status (*e.g.*, body weight gain and food intake) was evaluated during the exposure to the metals. Specifically, we measured the content of Zn and Cd in the brain of rats after *in vivo* exposure to metals. Furthermore, we evaluated the lipid peroxidation rate, the levels of MT, and the δ-ALA-D activity.

## 2 Materials and methods

### 2.1 Chemicals

δ-aminolevulinic acid (δ-ALA), DL-dithiothreitol (DTT), rabbit metallothionein-I, *o*-phenylendiamine, thiobarbituric acid (TBA) and malonaldehyde bis- (dimethyl acetal) were obtained from Sigma (St. Louis, MO, USA); mouse anti-metallothionein-I/II, and peroxidase-conjugated to goat anti-mouse IgG were purchased from Dako Corporation (Carpinteria, CA, USA).

### 2.2 Animals

Male Wistar rats (±80 days and ±240 g) were obtained from our animal facility. Animals were maintained under a controlled environment (three rats housed per cage, room temperature 22 °C, standard light/dark cycle of 12 h, and water and food provided *ad libitum*). Animal care was followed in accordance with the “National Institutes of Health Guide for Care and Use of Laboratory Animals”, and all experiments were approved by Ethics Committee of Universidade Federal do Rio Grande do Sul.

### 2.3 Treatments and tissue preparation

The rats (*n* = 9 animals per group) were daily exposed to Cd and/or Zn during 10 days. The metals were injected *via* intraperitoneal (i.p.), as a model for parenteral exposure of Cd, *i.e.*, inhalation [30]. Based on previous studies [16], [31], [32], animals were administered with neurotoxic doses of Cd in the presence or absence of supplementary dose of Zn. Experimental groups consisted in (i) NaCl 0.9% (control group), (ii) 2 mg kg^−1^ of Zn, (iii) 0.25 mg kg^−1^ of Cd, (iv) 0.25 mg kg^−1^ of Cd plus 2 mg kg^−1^ of Zn, (v) 1 mg kg^−1^ of Cd; (vi) 1 mg kg^−1^ of Cd plus 2 mg kg^−1^ of Zn. The metals were injected as acetate salts. Twenty-four hours after the last injection, the rats were anesthetized with sodium thiopental (40 mg/kg, 1 ml/kg, i.p.) and euthanized by decapitation. The whole brain was removed and separated in order to evaluate Zn and Cd levels and biochemical parameters. Importantly, the same brains were used to perform all tests. For biochemical analysis the brains were separately placed on ice and homogenized in saline (1:5) and centrifuged at 4000 g at 4 °C for 10 min. The remaining supernatant was used to determine the activity of the δ-ALA-D, MT content, and thiobarbituric acid-reactive substances (TBARS).

### 2.4 Body weight and food intake

The body weight gain of each animal was evaluated during exposure to the metals and the data were expressed as cumulative body weight gain per animal (*n* = 9 animals per group). The food intake per cage was also determined in each day of exposure to the metals, and the results were divided by number of animals in the cage. The food intake data were expressed as cumulative food intake (g) per animal (*n* = 3 independent experiments per group).

### 2.5 Zn and Cd determination

The content of Zn and Cd was determined by atomic absorption spectrometry following the US EPA 3052 protocol [33]. All analyses were carried out at the Centro de Ecologia of Universidade Federal do Rio Grande do Sul. The samples were digested in closed vials with 9 ml of 65% nitric acid and 3 ml of 30% hydrogen peroxide in boiling water. Zinc content in the samples (control, *n* = 6; 2 mg kg^−1^ of Zn, *n* = 5; 0.25 mg kg^−1^ of Cd, *n* = 6; 0.25 mg kg^−1^ of Cd plus 2 mg kg^−1^ of Zn, *n* = 6; 1 mg kg^−1^ of Cd, *n* = 6; 1 mg kg^−1^ of Cd plus 2 mg kg^−1^ of Zn, *n* = 6) was determined by a Perkin-Elmer 3300 flame atomic absorption spectrometer and Cd levels (control, *n* = 5; 2 mg kg^−1^ of Zn, *n* = 3; 0.25 mg kg^−1^ of Cd, *n* = 6; 0.25 mg kg^−1^ of Cd plus 2 mg kg^−1^ of Zn, *n* = 5; 1 mg kg^−1^ of Cd, *n* = 5; 1 mg kg^−1^ of Cd plus 2 mg kg^−1^ of Zn, *n* = 4) were estimated with a Perkin-Elmer SIMAA-6000 graphite furnace atomic absorption spectrometer. The minimum detection was 0.495 and 0.008 μg/g for Zn and Cd, respectively. All analyzes were determined with ultrapure reagents to avoid sample contamination.

### 2.6 Thiobarbituric acid-reactive substances

The lipid peroxidation was measured in the samples (control, *n* = 6; 2 mg kg^−1^ of Zn, *n* = 5; 0.25 mg kg^−1^ of Cd, *n* = 6; 0.25 mg kg^−1^ of Cd plus 2 mg kg^−1^ of Zn, *n* = 8; 1 mg kg^−1^ of Cd, *n* = 6; 1 mg kg^−1^ of Cd plus 2 mg kg^−1^ of Zn, *n* = 6) by relatively non-specific TBARS method according to Draper and Hadley [34]. Briefly, 80 μl of homogenized samples (2 μg/μl protein) were precipitated with 160 μl of TCA (15%) and centrifuged at 4000 g for 10 min. The supernatants (60 μl) and standard malondialdehyde (MDA) solutions were transferred to 96 well microplate and each well was filled with deionized water in order to obtain the equal volume. Next, 100 μl of TBA (0.67%) was added in each well and the microplate was sealed for heating in boiling water bath at 90 °C for 30 min. The TBARS levels were determined at 532 ηm and the values were expressed as μmol MDA equivalents/mg protein.

### 2.7 Metallothionein content

Metallothionein-I and -II (MT) were measured by enzyme-linked immunoassay (control, *n* = 7; 2 mg kg^−1^ of Zn, *n* = 7; 0.25 mg kg^−1^ of Cd, *n* = 9; 0.25 mg kg^−1^ of Cd plus 2 mg kg^−1^ of Zn, *n* = 8; 1 mg kg^−1^ of Cd, *n* = 7; 1 mg kg^−1^ of Cd plus 2 mg kg^−1^ of Zn, *n* = 7) [35]. The MT-III was not analyzed, because this isoform is not sensitive to Cd [36]. The experiment was conducted in 96-well microtiter plates at room temperature, and MT-I was used as standard. The plates were incubated with samples and standard concentrations of MT for 1 h. Then, the plates were washed three times with phosphate buffered saline plus 0.05% Tween 20 (washing solution). A blocking solution (washing solution plus 1% bovine serum albumin) was applied for 30 min, and the plates were washed three times. Then, mouse anti-metallothionein-I/II was incubated for 30 min and washed three times. Afterwards, peroxidase-conjugated to goat anti-mouse was incubated for 30 min and washed three times. Finally, *o*-phenylendiamine was incubated in the dark for 30 min for colorimetric reaction. The reaction was stopped with 3 M HCl and the plate was read at 490 ηm. The values were expressed as μg MT/mg protein.

### 2.8 δ-ALA-D activity

The enzyme activity (control, *n* = 5; 2 mg kg^−1^ of Zn, *n* = 7; 0.25 mg kg^−1^ of Cd, *n* = 7; 0.25 mg kg^−1^ of Cd plus 2 mg kg^−1^ of Zn, *n* = 5; 1 mg kg^−1^ of Cd, *n* = 7; 1 mg kg^−1^ of Cd plus 2 mg kg^−1^ of Zn, *n* = 7) was evaluated as previously described [37]. The formation rate of porphobilinogen (PBG) was obtained in medium containing 3 mM δ-ALA, 80 mM sodium phosphate buffer (pH 6.4) and brain supernatant (100 μg of protein per assay). To obtain δ-ALA-D reactivation (*n* = 5–7), DTT (2 mM) was also added to the medium in additional set of tubes as reported by Perottoni et al. [38]. The reaction was performed at 37 °C for 2 h, and PBG was determined using Ehrlich’s reagent at 555 ηm, with a molar absorption coefficient of 6.1 × 10^4^ M^−1^ for the Ehrlich-PBG salt.

### 2.9 Protein concentration

The protein content was estimated according to Lowry et al. [39].

### 2.10 Statistical analysis

Statistical results were obtained by STATISTICA/w 5.0 software and reported as mean ± S.E.M. The body weight gain and food intake were analyzed by two-way repeated-measures ANOVA, using days, Cd and Zn as factors. The neurochemical data were evaluated by two-way ANOVA, using Cd and Zn as factors. *Post hoc* comparisons were performed using Duncan’s multiple range test, and *p* < 0.05 was considered statistically significant.

## 3 Results

### 3.1 Body weight and food intake

**Fig. 1:**
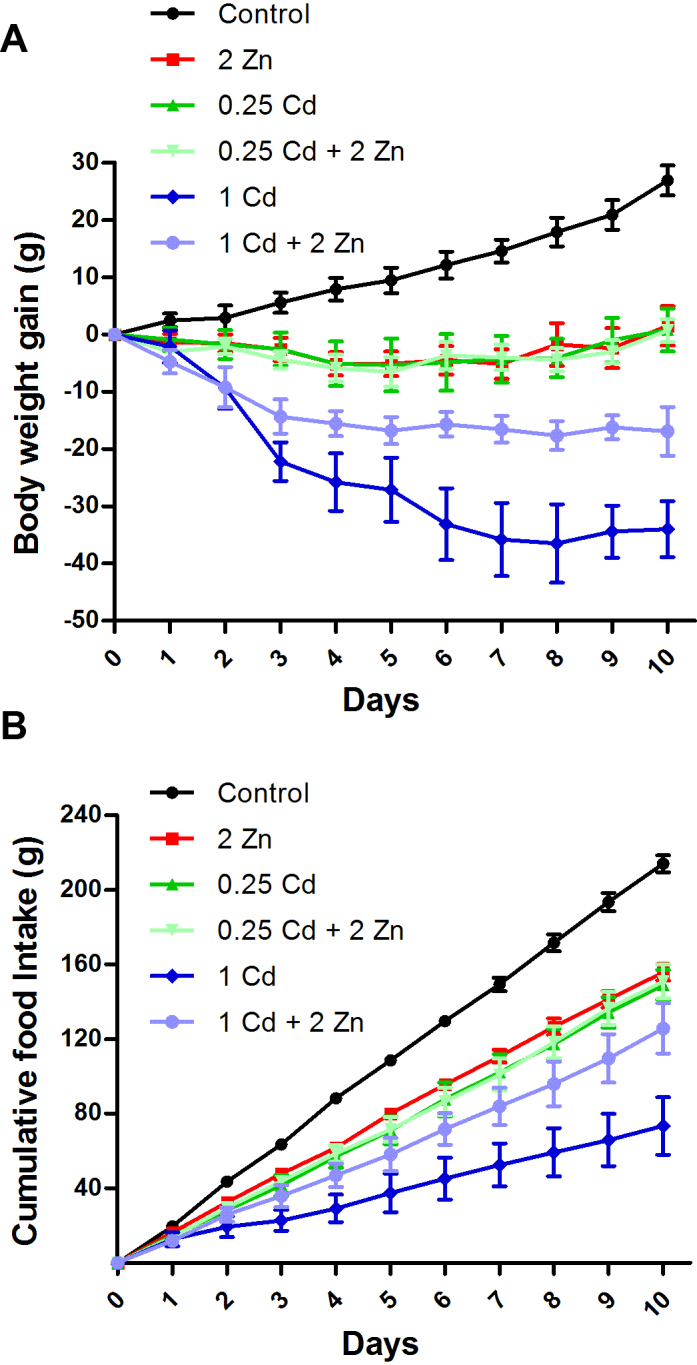
Animal status. The cumulative body weight gain (A) and the cumulative food intake (B) are represented during exposure to the metals. The lines indicate the results for control (black), 2 mg kg^−1^ of Zn (red), 0.25 mg kg^−1^ of Cd (green), 0.25 mg kg^−1^ of Cd plus 2 mg kg^−1^ of Zn (faint green), 1 mg kg^−1^ of Cd (blue), and 1 mg kg^−1^ of Cd plus 2 mg kg^−1^ of Zn (faint blue). Values are expressed as mean ± S.E.M. Data were analyzed by two-way repeated-measures ANOVA followed by Duncan’s multiple range *post hoc* test. Different letters indicate statistical differences at *p* < 0.05. (For interpretation of the references to color in this figure legend, the reader is referred to the web version of this article.)

Statistical analysis by two-way repeated-measures ANOVA indicated a significant effect of Cd (*F*[2,46] *=* 22.7*; p *< *0.001*), days (*F*[9,414] *=* 10.4; *p* < 0.001), Zn × Cd interaction (*F*[2,46] *=* 9.8; *p* < 0.001), Cd × days interaction (*F*[18,414] *=* 16.3; *p* < 0.001), and Zn × Cd × days interaction (*F*[18,414] *=* 10.5; *p* < 0.001) on body weight gain (Fig. 1A). Based on the initial and final weight difference of each animal, the control group showed a significant mean weight gain (25.4 ± 2.6 g) during the trial period, while Zn (0.4 ± 3.2 g), 0.25 mg kg^−1^ of Cd (0.8 ± 3.8 g) and 0.25 mg kg^−1^ of Cd plus Zn (1.1 ± 1.9 g) did not show significant increase in body weight after treatment period. The administration of 1 mg kg^−1^ of Cd caused a significant decrease in the body weight of rats (−34.6 ± 3.8 g). However, co-administration of Zn attenuated the Cd-induced body weight loss (−10.8 ± 4.2 g).

The analysis across 1-day intervals (Fig. 1B) by two-way repeated-measures ANOVA indicated a significant effect of Cd (*F*[2,12] *=* 27.5; *p* < 0.001), days (*F*[9,108] *=* 1178.2; *p* < 0.001), Zn × Cd interaction (*F*[2,12] *=* 9.3; *p* < 0.01), Cd × days interaction (*F*[18,108] *=* 38.7; *p* < 0.001), and Zn × Cd × days interaction (*F*[18,108] *=* 15.4; *p* < 0.001) on food intake. After 10 days of treatment, Zn (155.9 ± 4.5 g), 0.25 mg kg^−1^ of Cd (149.0 ± 8.0 g) and 0.25 mg kg^−1^ of Cd plus Zn (150.9 ± 9.2 g) did show a slight decrease in the food intake compared to the control group (214.0 ± 4.6 g). In contrast, the exposure to 1 mg kg^−1^ of Cd caused a significant decrease in the food intake of animals (73.4 ± 15.4 g), and the co-administration of Zn did attenuate this Cd effect (125.8 ± 13.5 g). In addition, co-administration of Zn also abolished the mortality caused by 1 mg kg^−1^ of Cd (∼22%).

### 3.2 Zn and Cd content

Two-way ANOVA showed only a significant effect of Cd in the accumulation of cerebral Cd (*F*[2,22] *=* 52.16; *p* < 0.001). The treatments did not modify the levels of Zn in the brain of rats (Table 1). Cadmium was not detected in the brains of the control and the Zn-treated group. However, Cd administration increased the levels of Cd in the brain and the accumulation was dose-dependent. Accordingly, treatment with 1 mg kg^−1^ of Cd did induce higher accumulation of Cd in the brain compared to 0.25 mg kg^−1^ of Cd. Cadmium accumulation was not modified by co-administration of Zn (Table 1).

**Table 1:** Zinc and cadmium concentrations in nervous tissue.

Groups	Zn*	Cd#
Control	14.02 ± 0.31	ND^a^
2 mg kg^−1^Zn	13.72 ± 0.22	ND^a^
0.25 mg kg^−1^Cd	14.18 ± 0.63	122.27 ± 22.16^b^
0.25 mg kg^−1^Cd + 2 mg kg^−1^Zn	13.22 ± 0.46	89.02 ± 21.01^b^
1 mg.kg^-1^Cd	13.07 ± 0.32	209.86 ± 20.86^c^
1 mg kg^−1^Cd + 2 mg kg^−1^Zn	13.29 ± 0.38	198.81 ± 17.72^c^

Different letters indicate statistical difference with *p* < 0.05. ND – not detectable.

* Data are presented as μg/g tissue, means ± S.E.M., *n* = 5–6.

# Data are presented as ηg/g tissue, means ± S.E.M., *n* = 3–6.

### 3.3 TBARS levels

**Fig. 2:**
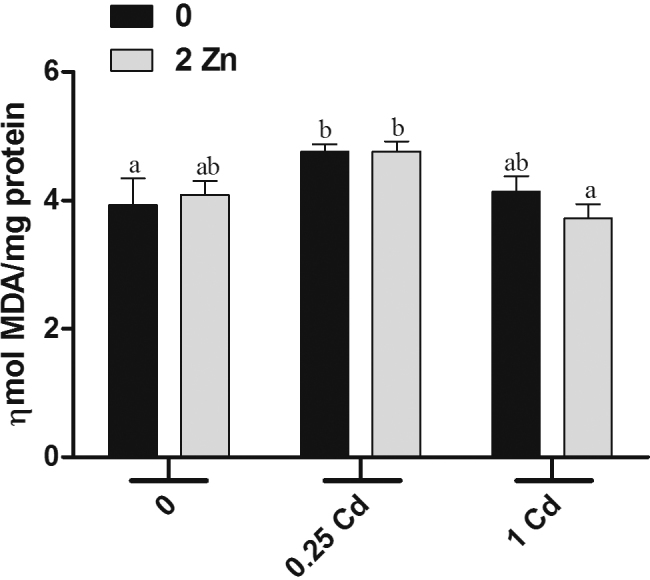
Brain concentration of TBARS after administrations with metals (*n* = 5–8). The gray and black bars indicate treatments with and without 2 mg kg^−1^ of Zn, respectively. The *x*-axis represents administrations with 0, 0.25, and 1 mg kg^−1^ of Cd. Values are expressed as mean ± S.E.M. Data were analyzed by two-way ANOVA followed by Duncan’s multiple range *post hoc* test. Different letters indicate statistical differences at *p* < 0.05.

The two-way ANOVA test showed only a significant effect of Cd on the TBARS levels (*F*[2,31] *=* 7.53; *p* < 0.01) (Fig. 2). The administration of Zn, 1 mg kg^−1^ of Cd and 1 mg kg^−1^ of Cd plus Zn caused no effect on TBARS. In contrast, the animals showed a significant increase in TBARS content after treatment with 0.25 mg kg^−1^ of Cd or 0.25 mg kg^−1^ of Cd plus Zn.

### 3.4 MT levels

**Fig. 3:**
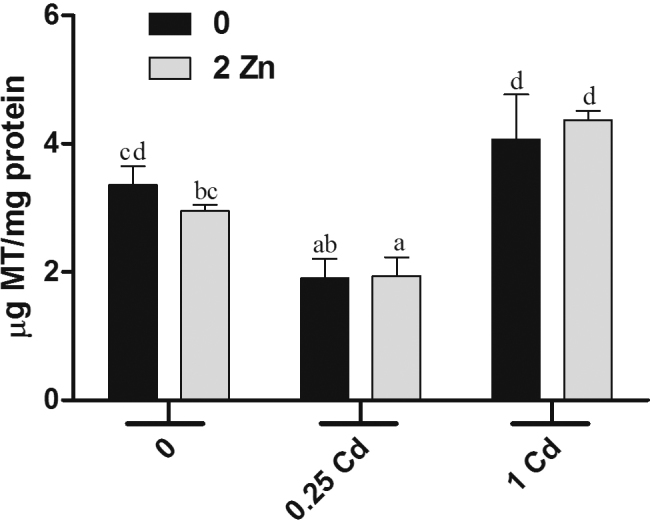
Brain concentration of MT after treatments with metals (*n* = 7–9). The gray and black bars indicate treatments with and without 2 mg kg^−1^ of Zn, respectively. The *x*-axis represents administrations with 0, 0.25, and 1 mg kg^−1^ of Cd. Values are expressed as mean ± S.E.M. Data were analyzed by two-way ANOVA followed by Duncan’s multiple range *post hoc* test. Different letters indicate statistical differences at *p* < 0.05.

The statistical analysis indicated only a significant effect of Cd in MT levels (*F*[2,39] *=* 21.98; *p* < 0.001). The MT levels are depicted in Fig. 3. The groups administered with 0.25 mg kg^−1^ of Cd and 0.25 mg kg^−1^ of Cd plus Zn showed significant reduction in amount of MT in the brain, while 1 mg kg^−1^ of Cd (with or without Zn) did not change the content of MT. Moreover, Zn treatment did not modify MT content, and Zn co-administration did not interfere also in Cd effects in MT levels.

### 3.5 δ-ALA-D activity

The two-way ANOVA analysis for activity and reactivation of δ-ALA-D also yielded no significant effect. The cerebral δ-ALA-D activities were not modified in treatments with Zn and/or Cd (Table 2). Similarly, the evaluation of enzyme reactivation indicated no change in this parameter (Table 2).

**Table 2:** Specific activity and reactivation index of δ-ALA-D in nervous tissue.

Groups	Specific activity*	Reactivation#
Control	24.0 ± 3.4	10.3 ± 1.5
2 mg kg^−1^Zn	24.3 ± 2.0	11.4 ± 2.9
0.25 mg kg^−1^Cd	19.9 ± 1.8	12.8 ± 1.3
0.25 mg kg^−1^Cd + 2 mg kg^−1^ Zn	23.2 ± 1.5	10.4 ± 0.8
1 mg kg^−1^Cd	22.3 ± 1.8	13.8 ± 2.4
1 mg kg^−1^Cd + 2 mg kg^−1^Zn	23.9 ± 1.3	11.3 ± 1.6

* Data are presented as ρmol PBG/min/mg protein, means ± S.E.M., *n* = 5–7.

# Data are presented as percentage of basal δ-ALA-D activity augment, means ± S.E.M., *n* = 5–7.

## 4 Discussion

The interaction between Cd and Zn has been studied in the last decade [16]. The interest in Cd–Zn interaction is due to Cd be an environmental pollutant with potential action in Zn-dependent pathways in biological systems. The exposure to Cd can modify Zn homeostasis by inducing the transport of Zn to target organs such as liver and kidneys [16]. However, Zn can blunt or reverse the toxicity of Cd [16]. In previous study, we have also reported that Zn protected against mortality and loss of body weight caused by Cd, and these results were associated to a mitigating effect of Zn against Cd-induced changes in MT levels in the liver of rats [27]. In the present work, we have investigated whether the interaction between these metals could also be extended to the brain. Here, we observed that the mortality, body weight loss, and food intake deprivation caused by subacute administration of Cd was associated to cerebral accumulation of Cd, but Zn levels were not altered in the nervous tissue of Cd-treated rats. The Zn-dependent activity of brain δ-ALA-D was not also modified after treatments with Cd and/or Zn. However, the treatment with 0.25 mg kg^−1^ of Cd caused a decrease in MT levels and a stimulation in the lipid peroxidation in the brain. Finally, the co-administration of Zn did not attenuate any effect induced by Cd in the brain.

It is well known that Zn is an essential metal in the body, where presents cofactor function on important enzymes [40]. In addition, Zn has special role in the brain of vertebrates, because it is also a neuromodulator in excitatory synapses [26]. In contrast to Zn, Cd is a toxic metal that scarcely reaches the brain under normal conditions due to the presence of the blood brain barrier (BBB) [41]. However, our results clearly demonstrate that i.p. administration of Cd resulted in accumulation of this metal in the brain. Thus, this change could indicate a possible alteration in cerebral Zn content, since it has been reported a modification in tissue levels of Zn in Cd target organs [16]. Nevertheless, we observed no modification in cerebral Zn caused by Cd accumulation, such as demonstrated in previous studies [28], [29], [30]. Moreover, our data also showed that Zn co-administration was unable to alter Zn and Cd levels in the brain. These results could indicate that brain responds differently to exposure to Cd in comparison to other tissues. However, a previous study has reported that Zn pre-treatment promoted simultaneously a lower and higher accumulation of Cd in the brain and liver, respectively [42]. Thus, it is possible that cerebral accumulation of Cd was not enough to trigger an increase in Zn content as observed previously in the liver.

The co-administration of Zn did cause no effect in the cerebral accumulation of Cd, but the protective action of Zn on harmful effects caused by Cd in animals status could be explained for a neurochemical action of these metals. In the current study, we have evaluated TBARS and MT content, because Cd has known action on both biochemical endpoints [7], [22]. Although, we obtained a considerable background in TBARS technique by non-specific reactions [43], [44], [45], [46], which could have underestimated lipid peroxidation, our results showed that exposure to lowest dose of Cd did increase lipid damages as previously reported [47]. Interestingly, we observed that these effects were related to reduction in MT levels. Since MT presents Cd chelating properties as well as scavenging ability of reactive oxygen species [48], [49], [50], [51], it is supposed that lipid damage performed by 0.25 mg kg^−1^ of Cd did occur as consequence of low levels of MT. This hypothesis is further supported by administration of 1 mg kg^−1^ of Cd, which did not change lipid peroxidation rate and MT levels. Consequently, it could be expected a protective mechanism of Zn involving overexpression of MT. However, it was noted that co-administration of Zn was not able to modify the effects of Cd on lipid peroxidation and MT levels in the brain. There are scarce data about the evaluation of these parameters in the brain after Cd and Zn exposure, but it has been reported that the methamphetamine-induced lipid peroxidation was attenuated by an increment on MT levels in dopaminergic cells after Zn pretreatment [52], [53]. Moreover, *in vivo* studies showed an increase in transcriptional expression of MT caused by intracerebroventricular injection of Zn [54], [55]. In fact, these studies indicate the similar ability of Zn as inducer of MT expression in the brain, such as observed in other tissues [22]. As we observed no changes on total Zn content in the brain, the administration of 2 mg kg^−1^ of Zn by i.p. route, as we performed, was possibly insufficient to modulate MT content and lipid peroxidation in the nervous tissue. Therefore, it is suggested that the protective mechanism of Zn on effects of Cd in animal status is not related to lipid peroxidation and MT levels in the brain.

An important role of the MT is the control of cellular levels of Zn [48], [50]. Indeed, the change in MT levels caused by Cd could affect the function of Zn-dependent biomolecules. In order to obtain data about this hypothesis, we evaluated the thiol- and Zn-dependent acitivity of δ-ALA-D [21], [37]. Based on our results, the cerebral enzyme function was preserved in any of the experimental groups, including those animals with low levels of MT. Other works have reported also no change in brain δ-ALA-D activity of rodents after exposure to Cd [24], [56], [57]. Since the δ-ALA-D is one of the enzymes most sensitive to cellular levels of Zn and Cd [58], [59], all these results support that the Zn-dependent activities were probably little affected in the brain after Zn and Cd treatments.

In the present study, the animal status showed an attenuating effect of Zn on Cd toxicity, but Zn administration did not modify any action performed by Cd in neurochemical parameters. Interestingly, these data indicate a protective Zn mechanism weakly related to the brain. However, this does not exclude fully the involvement of the brain on mitigatory function of Zn on Cd. Indeed, other potential molecules as well as specific cerebral structures may be a target of Zn–Cd interaction in the brain. For instance, calcium (Ca) uptake [60] and the expression of Ca transporter-1 [61] are deeply modulated by Cd and Zn in the kidney tissue and these same Ca molecular components could be sensitive to interactive effect of Zn and Cd in the brain. In addition, some structural brain parts could be differently affected for both metals, since a small fraction of total Zn brain, the chelatable Zn pool, is heterogeneously distributed in the CNS [26]. In this case, the structures with high and/or low levels of chelatable Zn could present an interactive effect of Cd and Zn, which we did not observe in the whole brain. Thus, future additional studies, investigating Zn–Cd interaction in Ca-related molecules and chelatable Zn of brain structures, can complement our findings on total Zn content in the whole brain.

In summary, this is the first study considering multiple exposures of Cd and Zn on these Zn-dependent pathways. Under a whole brain approach, we demonstrated that the brain is also subject to Cd action, leading to lipid damage associated to reduction in the levels of MT. However, in contrast to liver, Zn treatment did not modify the cerebral effects caused by Cd. Therefore, these data strongly indicate that the protective effect of Zn on impairments induced by Cd in animal status is predominantly restricted to molecular mechanisms at peripheral tissues, because these organs are possibly most susceptible to Cd accumulation than the brain.

## Funding

This study was supported by FINEP research grant “Rede Instituto Brasileiro de Neurosciência (IBN-Net)” # 01.06.084200. We also express our special thanks to Prof. Dr. Tuiskon Dick (1927–2008) by the guidance of this work and mainly for his contribution to Brazilian Science.

## Conflict of interest

None declared.
